# Molecular heterogeneity of CD30+ diffuse large B-cell lymphoma with prognostic significance and therapeutic implication

**DOI:** 10.1038/s41408-022-00644-2

**Published:** 2022-03-29

**Authors:** Yu-Jia Huo, Peng-Peng Xu, Di Fu, Hong-Mei Yi, Yao-Hui Huang, Li Wang, Nan Wang, Meng-Meng Ji, Qing-Xiao Liu, Qing Shi, Shuo Wang, Shu Cheng, Yan Feng, Wei-Li Zhao

**Affiliations:** 1 Shanghai Institute of Hematology, State Key Laboratory of Medical Genomics, National Research Center for Translational Medicine at Shanghai, Ruijin Hospital, Shanghai Jiao Tong University School of Medicine, Shanghai, China; 2 Department of Pathology, Shanghai Ruijin Hospital, Shanghai Jiao Tong University School of Medicine, Shanghai, China; 3 Pôle de Recherches Sino-Français en Science du Vivant et Génomique, Laboratory of Molecular Pathology, Shanghai, China; 4 State Key Laboratory of Microbial Metabolism, School of Life Sciences and Biotechnology, Shanghai Jiao Tong University, Shanghai, China

**Keywords:** Cancer genetics, B-cell lymphoma

**Dear Editor**,

Diffuse large B-cell lymphoma (DLBCL) is the most common subtype of non-Hodgkin lymphoma with heterogeneous clinical, immunophenotypic, and genetic features. Surface antigens, such as CD20, CD22, CD79B, and CD19 are important accessible parts of lymphoma cells with immunotherapeutic potential. CD30, encoded by *TNFRSF8*, is a transmembrane cytokine receptor of the tumor necrosis factor receptor (TNFR) superfamily and expressed on ~20% of DLBCL. To better understand the underlying mechanism of CD30 expression in DLBCL, we performed clinical, genomic and transcriptomic analysis in a cohort of 1048 patients with de novo DLBCL. A flow chart describing the cohort selection was displayed in Supplementary Fig. 1a. CD30 expression examined by immunohistochemistry (IHC) and by RNA sequencing were highly correlated (*P* < 0.001, Supplementary Fig. 1b). Using semi-quantitative IHC, 118 cases showed CD30 over 20% (including 86 cases over 50%) and 135 cases showed CD30 ranged from 1% to 20%, which accounted for 11.3% and 12.9% of DLBCL, respectively (Supplementary Fig. 1c). The main clinical and pathological characteristics according to CD30 expression were summarized in Supplementary Table 1. CD30 + DLBCL had elevated serum lactic dehydrogenase (*P* = 0.002), as compared to CD30-DLBCL. In terms of IPI, 39% of CD30 + DLBCL and 48% of CD30-DLBCL were categorized into low-risk group, 21% and 19% into low-intermediate risk group, 25% and 17% into intermediate-high risk group, and 16% and 16% into high-risk group, respectively (*P* = 0.040). Pathologically, CD30 + DLBCL was significantly associated with increased percentage of non-GCB subtype (*P* = 0.042) and EBER positivity (*P* < 0.001), as previously reported [1]. Clinical and pathological characteristics of CD30 + DLBCL were similarly distributed between cases with CD30 over 20% and with CD30 ranged from 1% to 20% (Supplementary Table 2). Upon R-CHOP treatment, with median follow-up of 38.7 months, the 5-year progression-free survival (PFS) and overall survival (OS) rates of CD30 + DLBCL were 61.4% and 72.4%, similar as those of CD30-DLBCL (Supplementary Fig. 2). To our knowledge, this is the largest integrated clinical, pathological, genomic, and transcriptomic analysis of CD30 + DLBCL.

Gene mutations were screened in 636 patients, including 172 CD30 + DLBCL and 464 CD30-DLBCL. CD30 + DLBCL had a higher mutation frequency in *TNFAIP3* (*P* = 0.002), *TNFRSF14* (*P* = 0.045), *SOCS1* (*P* = 0.010), *STAT6* (*P* = 0.007), *CIITA* (*P* = 0.007), *CD58* (*P* = 0.037), *KMT2C* (*P* = 0.016), but lower mutation frequency in *CD79B* (*P* = 0.026) and *MYD88* (*P* = 0.010, Fig. 1A). Differential mutations annotated by pathways were summarized in Fig. 1B. RNA sequencing was also screened in 385 patients, including 117 CD30 + DLBCL and 268 CD30-DLBCL. CD30 + DLBCL showed significant enrichment in oncogenic pathways such as NF-κB, JAK-STAT, NOTCH, MAPK, and immune-associated pathways, including cell adhesion molecules, toll-like receptor, TNF-mediated, antigen processing and presentation, nod-like receptor, and T-cell receptor pathway (Fig. 1C), in consistent with alterations in other CD30 + lymphomas like Hodgkin lymphoma (HL) and primary mediastinal large B-cell lymphoma [2, 3]. Moreover, tumor microenvironment in CD30 + DLBCL was analyzed by RNA sequencing. T-cell activation score and immune escape score were used as two parameters to determine the overall immune status [4]. CD30 + DLBCL showed higher score in both T-cell activation and immune escape than CD30-DLBCL (*P* = 0.004 and *P* < 0.001, Fig. 1D). Infiltration of immune cell subtypes were further assessed. CD30 + DLBCL was significantly associated with increased infiltration of CD4 + T-cells (*P* = 0.020), cytotoxic T-cells (*P* = 0.030), exhausted T-cells (*P* = 0.004), T-helper 17 (Th17) cells (*P* = 0.001), regulatory T (Treg)-cells (*P* = 0.001), dendritic cells (DC, *P* < 0.001), and macrophages (*P* = 0.001, Fig. 1E), indicating a “hot” tumor with infiltrations of various immune cells. Together, these results suggested that CD30 + DLBCL may reflect a specific entity with unique molecular and microenvironment features.

**Fig. 1:**
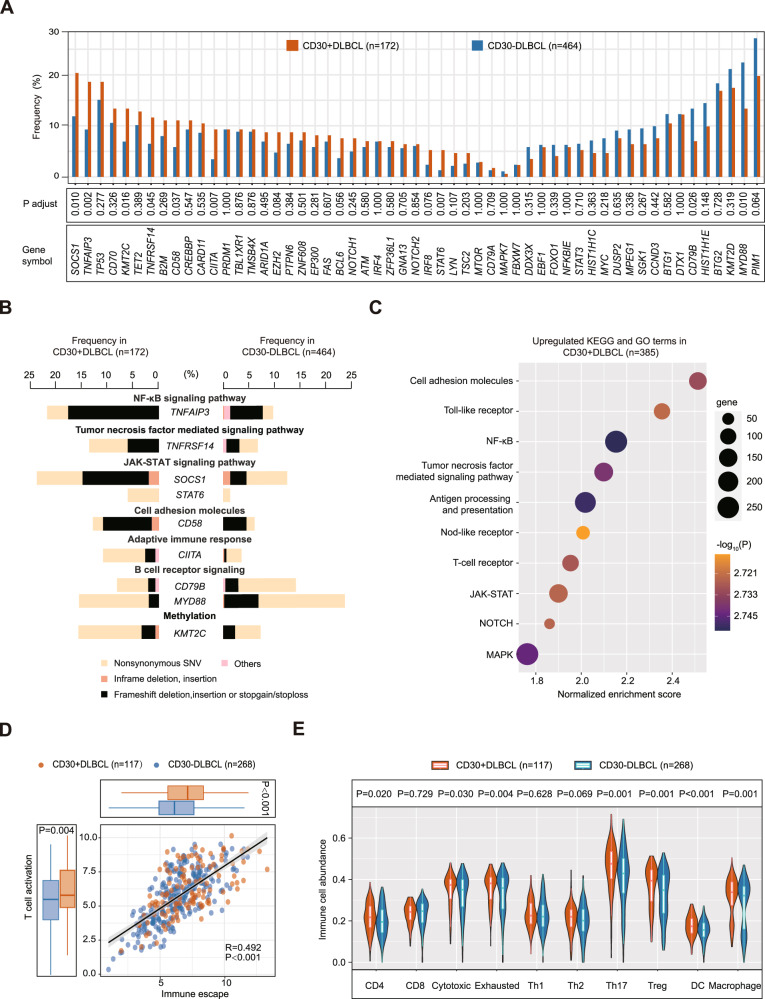
Genomic and transcriptome analysis in DLBCL according to tumor CD30 expression. **A** Prevalence of gene mutations in 55 lymphoma-associated genes, along with the P value for the difference in prevalence between 172 CD30 + DLBCL and 464 CD30-DLBCL. **B** Prevalence and type of recurrent mutations and their function in CD30 + DLBCL. Mutation types are color-coded as indicated in the legend below. **C** Gene-Set Enrichment Analysis (GSEA) revealed pathway alterations in 117 CD30 + DLBCL and 268 CD30-DLBCL. GSEA preranked tool was used to analyze oncogenic and immune-associated pathways. **D** Left and upper panels indicate T-cell activation score and immune escape score, respectively, stratified by 117 CD30 + DLBCL and 268 CD30-DLBCL. **E** Infiltration score of indicated immune cells in tumor samples according to CD30 expression.

Based on correlation between gene mutations and signaling pathways, we defined three molecular subtypes of CD30 + DLBCL (Supplementary Fig. 3a, b and supplementary Method). *TNFAIP3*, *SOCS1* and *CIITA* mutations represented three major molecular alterations in CD30 + DLBCL. Genetic composition of each subtype was displayed, while selected genes had the highest mutation frequency in corresponding subtype (Fig. 2A). Molecular subtypes were established, including NM subtype (61 cases, based on mutations in TNFR-related NF-κB pathway and methylation), JA subtype (56 cases, based on mutations in JAK-STAT pathway and acetylation) and IB subtype (45 cases, based on mutations in immune modulation and B-cell receptor (BCR)-MAPK pathway). Ten CD30 + DLBCL patients were not classified into either subtype due to absence of relevant mutations. No significant difference was observed among three subtypes according to CD30 expression estimated by IHC or RNA sequencing, nor according to clinical and pathological characteristics (Supplementary Fig. 3c–e and supplementary Table 3). Of note, upon R-CHOP treatment, three molecular subtypes differed significantly in PFS and OS (*P* = 0.049 and *P* = 0.020), with worst prognosis in JA subtype (Fig. 2B). In univariate and multivariate analysis, JA subtype was an independent predictor for inferior PFS and OS (Supplementary Table 4). Oncogenic signaling, immune modulation, and epigenetic process were differentially manifested in molecular subtypes (Fig. 2C and Supplementary Fig. 4a). The NM subtype mainly involved activation of NF-κB pathway and hypermethylation process. For example, *TNFAIP3* and *TNFRSF14* mutations lead to NF-κB activation and constitutive CD30 expression [5]. *KMT2D* and *KMT2C* mutations dysregulate H3K4 methylation, resulting in NF-κB activation [6]. The JA subtype mainly involved activation of JAK-STAT pathway and deacetylation process. JAK-STAT pathway genes, including *SOCS1* and *STAT6*, are recurrently mutated in CD30 + lymphoma [2, 3]. *CREBBP* and *EP300* mutations inhibit H3K27 acetylation, contributing to activation of JAK-STAT and NOTCH pathway [7, 8]. The IB subtype mainly involved alterations in immune modulation, including inhibition of antigen possessing and presentation and activation of BCR-MAPK pathway. Genetic alterations in *CIITA* and *B2M* induce immune privilege and define the main characteristics of CD30 + lymphomas [2, 3]. BCR pathway is implicated in multiple aspects of immune modulation and RAS-MAPK pathway acts as downstream pathway of BCR [9].

**Fig. 2:**
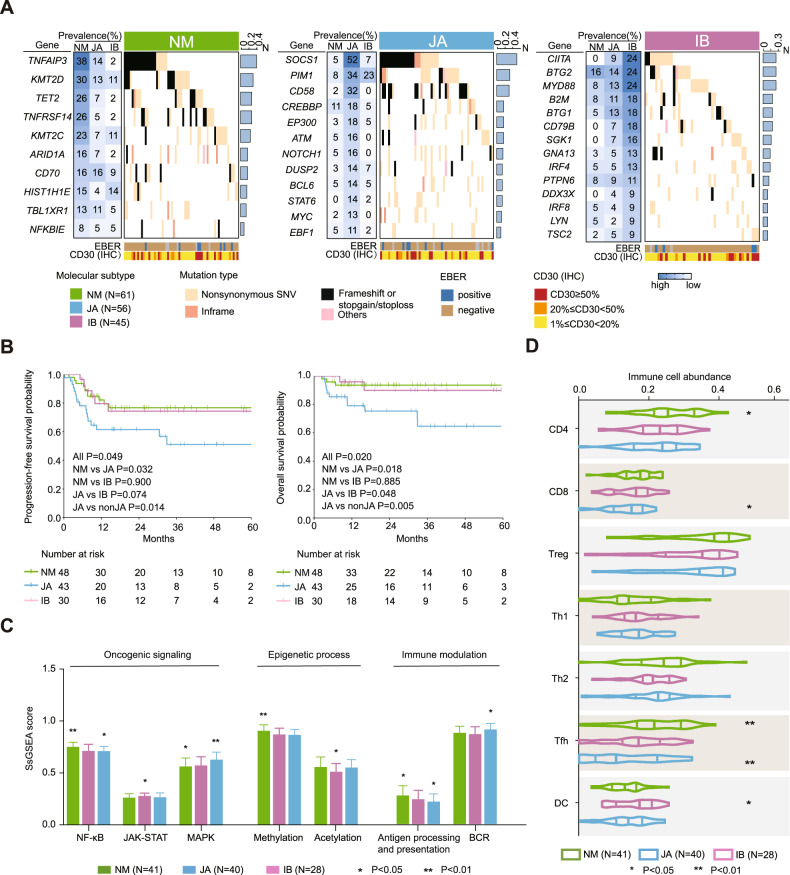
CD30 + DLBCL molecular subtypes with prognostic significance and therapeutic implication. **A** Genetic composition of CD30 + DLBCL molecular subtypes. The prevalence of the indicated genetic features was indicated at the left. Column represent each DLBCL patient with information of CD30 expression estimated by immunohistochemistry and EBER. **B** Kaplan–Meier curves for progression-free survival (left panel) and overall survival (right panel) according to three molecular subtypes. **C** Single sample Gene-Set enrichment analysis (ssGSEA) score of signature genes in each molecular subtype. Top graph shows annotated pathways into three major classes (oncogenic signaling, epigenetic process and immune modulation). *P* value was calculated between certain molecular subtype versus other subtypes of CD30 + DLBCL. Error bars denote SD. **D** Infiltration score of indicated immune cells in each molecular subtype. *P* value was calculated between certain molecular subtype versus other subtypes of CD30 + DLBCL.

As for tumor microenvironment, the NM subtype presented higher CD4 + T (*P* = 0.020) and follicular helper T (Tfh) cells (*P* = 0.001), with a positive correlation between the signature genes for immune cell recruitment such as CCR6, CCL21, and CXCR4 (all *P* < 0.001, Fig. 2D and supplementary Fig. 4b). NF-κB pathway may promote germinal center formation with the help of CD4 + Tfh by the above chemokines, contributing to activation of immune response [10]. The JA subtype presented higher DC infiltration (*P* = 0.035), in parallel with immune checkpoint genes, such as PD-L1, TIM3, and LAG3 (all *P* < 0.001, Fig. 2D and supplementary Fig. 4b). Activation of interferon-JAK-STAT axis upregulates expression of these immune checkpoints in tumor immunity, resulting in increased exhausted DCs and immune tolerance [11, 12]. The IB subtype presented lower Tfh (*P* = 0.007) and CD8 + T-cells (*P* = 0.049, Fig. 2D). Loss of antigen processing and presentation results in low immune cell infiltration of this subtype [2].

Preclinical models mimicking three molecular subtypes were subsequently constructed. Mutational sites of *TNFAIP3, SOCS1* and *CIITA* were illustrated in Supplementary Fig. 5a. Based on expression of *TNFAIP3*/*SOCS1*/*CIITA* in OCI-LY10 and SU-DHL-4 cells (Supplementary Fig. 5b), *TNFAIP3*^wt^, *TNFAIP3*^L147Q^, as well as a shRNA to knockdown *SOCS1* (*SOCS1*^kd^) and *CIITA* (*CIITA*^kd^), were transfected into OCI-LY10. Meanwhile, *SOCS1*^wt^, *SOCS1*^Q175H^, *CIITA*^wt^, *CIITA*^L807R^, as well as a shRNA to knockdown *TNFAIP3* (*TNFAIP3*^kd^), were transfected into SU-DHL-4. Quantitative real-time PCR and western blot were used to confirm the transfection efficiency (Supplementary Fig. 5c, d). CD30 expression was upregulated in *TNFAIP3*^kd^, as compared to scramble, with similar pattern in *SOCS1*^Q175H^ to *SOCS1*^wt^, and *CIITA*^L807R^ to *CIITA*^wt^ in OCI-LY10 (Supplementary Fig. 6a). Accordingly, CD30 expression was upregulated in *TNFAIP3*^L147Q^, *SOCS1*^kd^, *CIITA*^kd^, as compared to *TNFAIP3*^wt^ and scramble in SU-DHL-4 (Supplementary Fig. 6b). Therefore, our data demonstrated that function loss of *TNFAIP3*/*SOCS1*/*CIITA* increased CD30 expression and corresponded to three molecular subtypes of CD30 + DLBCL. Brentuximab vedotin (BV), an anti-CD30 monoclonal antibody-drug conjugate has shown remarkable responses in first-line treatment of HL and CD30 + peripheral T-cell lymphoma in combination with chemotherapy [13]. In NM model, increased sensitivity to BV was shown in *TNFAIP3*^L147Q^ versus *TNFAIP3*^wt^ OCI-LY10 (*P* = 0.019 for 96 h half maximal inhibitory concentration [IC50]), and *TNFAIP3*^kd^ versus scramble SU-DHL-4 (*P* = 0.009 for 96 h IC50, Supplementary Fig. 6c). In JA model, increased sensitivity to BV was shown in *SOCS1*^kd^ versus scramble OCI-LY10 (*P* = 0.010 for 96 h IC50), and *SOCS1*^Q175H^ versus *SOCS1*^wt^ SU-DHL-4 (*P* = 0.003 for 96 h IC50, Supplementary Fig. 6d). In IB model, increased sensitivity to BV was shown in *CIITA*^kd^ versus scramble OCI-LY10 (*P* = 0.020 for 96 h IC50), and *CIITA*^L807R^ versus *CIITA*^wt^ SU-DHL-4 (*P* < 0.001 for 96 h IC50, Supplementary Fig. 6e). Based on in vitro data that BV enhanced efficacy in three molecular subtypes of CD30 + DLBCL, we supposed that BV was able to kill CD30 + tumor cells of DLBCL. Indeed, a phase II study evaluated the efficacy of BV in relapsed/refractory patients and demonstrated 44% objective response rate and 15% complete response rate (NCT01421667), in which responses occurred in DLBCL patients independent on CD30 expression (>1%) [14]. In the R-CHOP era, rituximab can prolong the survival of NM subtype characterized by NF-κB activation, and IB subtype characterized by BCR-MAPK activation as well [15]. However, JA subtype characterized by JAK-STAT activation showed poor survival and may be potential candidate for BV treatment. Alternatively, with better understanding of underlying mechanism of CD30 expression, targeted agents may also be important adjunctive strategies, such as epigenetic-targeted histone deacetylase inhibitors, JAK-STAT inhibitors, and BTK inhibitors, etc.

In conclusion, CD30 + DLBCL is a heterogenetic entity and differs significantly in clinical outcomes, gene mutations, transcriptional signatures, and tumor microenvironment patterns. Molecular subtypes with different genetic features may indicate potential efficacy of BV and guide future mechanism-based targeted therapy in DLBCL.

## Data sharing

Sequencing data is available at the National Omics Data Encyclopedia (NODE, https://www.biosino.org/node/ Project ID: OEP001143). There are currently no plans to share data not included in this paper.

## Supplementary information

Supplemental Materials
