# Supplementary material for: Molecular heterogeneity of CD30+ diffuse large B-cell lymphoma with prognostic significance and therapeutic implication
Source: Blood Cancer J. 2022 Mar 29;12(3):48. doi: 10.1038/s41408-022-00644-2 (PMC8964673; doi:10.1038/s41408-022-00644-2)
Supplement: Supplementary file 1 — Supplemental Materials [file 41408_2022_644_MOESM1_ESM.docx]

Supplementary Materials for

Molecular heterogeneity of CD30+ diffuse large B-cell lymphoma with prognostic significance and therapeutic implication

**Yu-Jia Huo^1†^, Peng-Peng Xu^1†^, Di Fu^1†^, Hong-Mei Yi^2†^, Yao-Hui Huang^1^, Li Wang^1,3^, Nan Wang^1^, Meng-Meng Ji^1^, Qing-Xiao Liu^2^, Qing Shi^1^, Shuo Wang^1^, Shu Cheng^1^, Yan Feng^4^, Wei-Li Zhao^1,3*^**

**^†^** Contributed equally

^1^ Shanghai Institute of Hematology, State Key Laboratory of Medical Genomics, National Research Center for Translational Medicine at Shanghai, Ruijin Hospital, Shanghai Jiao Tong University School of Medicine, Shanghai, China;

^2^ Department of Pathology, Shanghai Ruijin Hospital, Shanghai Jiao Tong University School of Medicine, Shanghai, China;

^3^ Pôle de Recherches Sino-Français en Science du Vivant et Génomique, Laboratory of Molecular Pathology, Shanghai, China;

^4^ State Key Laboratory of Microbial Metabolism, School of Life Sciences and Biotechnology, Shanghai Jiao Tong University, Shanghai, China.

* Correspondence to: Wei-Li Zhao, Shanghai Institute of Hematology, State Key Laboratory of Medical Genomics, National Research Center for Translational Medicine at Shanghai, Ruijin Hospital, Shanghai Jiao Tong University School of Medicine, 197 Rui Jin Er Road, Shanghai, 200025, China. Telephone: 0086 64370045, Email: [zhao.weili@yahoo.com](mailto:zhao.weili@yahoo.com).

**This PDF file includes:**

Supplementary Methods

Supplementary Figures. S1 to S6

Supplementary Tables. S1 to S9

**Supplementary Methods**

1. Study populations

We studied 1122 patients with de novo DLBCL based on registry data from January 2005 to December 2020 (supplementary Fig. 1a). Among 1122 patients with immunohistochemical results of CD30, 1048 patients were enrolled, excluding patients with primary central nervous system lymphoma (N=24), primary mediastinal large B-cell lymphoma (N=32­) or treated with supportive care (N=18). All patients were diagnosed according to World Health Organization (WHO) classification^1^. International prognostic index (IPI) was calculated, as previously described^2^. The study was approved by the Shanghai Ruijin Hospital Review Board with informed consent obtained in accordance with the Declaration of Helsinki.

2. Immunohistochemistry and fluorescence in situ hybridization analyses

Immunohistochemistry was performed on 5 μm paraffin sections using indirect immunoperoxidase method by antibodies against CD30, CD10, BCL6, MUM1, BCL2, MYC and EBER in-situ hybridization was performed on 3-μm-paraffin sections (supplementary Table 5). Protein expression levels of CD30 were scored based on percentage of stained cells semi-quantitatively, and the cutoff value for CD30 was 1%, as previously described^3,4^. Germinal center B-cell (GCB) or non-GCB subgroups were determined using Hans classification^5^, with 30% cutoff value of CD10, BCL6, and MUM1. For BCL2/MYC double expression (DE), the cutoff value of BCL2 and MYC were 50% and 40%, respectively^6^. Epstein-Barr virus-encoded RNA (EBER) was performed by in-situ hybridization (ISH) and the cutoff value was 20%^7^. Staining was not always achieved for each marker owing to tissue exhaustion. Two expert pathologists Hongmei Yi and Qingxiao Liu performed a blind revision of the samples. Fluorescence in situ hybridization was performed using fusion probe IGH/BCL2 (14q32/18q21), break-apart probes BCL6 (3q27) and C-MYC (8q24) (GP Medical Technologies, Ltd, Beijing, China) according to the guidelines of the manufacturer. At least 150 nuclei per sample were counted under a fluorescent microscope. The case was considered positive when more than 10% of the whole counted tumor cells showed break-apart or fusion signals. Double hit (DH) was defined as a MYC translocation and a concurrent translocation of BCL2 or BCL6 estimated by fluorescence in situ hybridization.

3. Sequencing protocols and bioinformatic principles

Genomic DNA was extracted from frozen or formalin-fixed paraffin-embedded (FFPE) tumor samples using a QIAamp DNA Mini Kit (Qiagen, Duesseldorf, Germany) or a QIAamp DNA FFPE Tissue Kit (Qiagen), respectively, based on the manufacturer’s guidelines. Matched peripheral blood DNA was extracted using a QIAamp DNA Mini Kit (Qiagen, Duesseldorf, Germany). Targeted sequencing (n=446), whole exome sequencing (WES, n=122), or whole genome sequencing (WGS, n=68) was performed in 636 patients with frozen or qualified formalin-fixed paraffin-embedded tumor samples. Among them, 195 targeted sequencing, 117 WES, and 68 WGS data were referred in our previous study^8^, and other data were newly added. WES (n=25, divided into five groups) and WGS (n=17) were performed on 42 matched peripheral blood samples randomly selected to build a somatic mutation calling principle and to exclude germ-line polymorphisms. Sanger sequencing was used to confirm somatic mutations not observed in matched peripheral blood mononuclear cells.

For 68 patients, WGS was performed on frozen tumor tissue. Genomic DNA concentrations were measured with the Qubit (Thermo Fisher Scientific), and sheared to about 300bp fragments by Covaris DNA shearing system. After end-repaired and 3’-ends adenylated, Illumina PE adapters were ligated to DNA fragments to generate indexed library. Library was validated by Agilent 2100 Bioanalyzer and sequencing was performed on Illumina HiSeq platform with 150bp paired-end strategy in WuXi NextCODE, Shanghai.

For 122 patients, WES was carried out on frozen tumor tissue (n=78), or on FFPE tumor tissue quality controlled by agarose gel electrophoresis (n=44). Exome regions were captured by a SeqCap EZ Human Exome kit (version 3.0) and sequencing was performed on HiSeq 4000 platform with 150bp paired-end strategy in Righton, Shanghai.

By Burrows-Wheeler Aligner (BWA) version 0.7.13-r1126, read pairs were aligned to RefSeq hg19 (downloaded from UCSC Genome Browser, URLs). Samtools version 1.3 was used to generate chromosomal coordinate-sorted bam files and to remove PCR duplications. The reads were then realigned around potential indel regions by Genome Analysis Toolkit (GATK) version 3.4 IndelRealigner with the recommended pipeline. Of each sample, the mean depth measured with WES/WGS was 120.25 × (range 50-200 ×), with an average 97.65% (range 82.64%-99.06%) of the target sequence being covered sufficiently deep for variant calling (≥ 10 × coverage).

GATK Haplotype Caller and GATK Unified Genotyper were applied to call SNVs and indels. Homemade pipeline was used to filter SNVs and indels detected by the above software, excluding: 1) mutations reported with low confidence; 2) germline mutations detected from control samples; 3) population-related variants reported in 1000 Genomes (dbSNP 137) as common SNPs and not included in COSMIC (the Catalogue of Somatic Mutations in Cancer) version v77. SNVs and indels were mapped to the genome location using the UCSC Genome Browser ([http://genome.ucsc.edu](http://genome.ucsc.edu/)). All the somatic functional mutations, including nonsynonymous SNVs, frameshift or in-frame indels, stopgain or stoploss and so on were obtained. Visual inspection was used to exclude potential false positive results.

For targeted sequencing, extended 446 patients with FFPE tumor tissue quality-controlled by agarose electrophoresis were included. Based on the WES and WGS results, we identified 55 recurrent and functional mutated genes for targeted sequencing^8^. PCR primers were designed by Primer 5.0 software. Multiplexed libraries of tagged amplicons from tumor tissue samples were generated by Shanghai Righton Bio-Pharmaceutical Multiplex-PCR Amplification System. Deep sequencing was performed using established Illumina protocols on HiSeq 4000 platform (Illumina). Sanger sequencing was used to confirm somatic mutations of 55 mutated genes.

4. Subtype establishment

As for classification, the first step was to confirm the “core seed gene” of each molecular subtype. Other genes that algorithmically correlated with specific “core seed gene” were also added into this subtype and called the “seed gene”. Next, patients who harbored the “seed gene” mutations were divided into each molecular subtype. Meanwhile, some of the remaining genes were added into each molecular subtype in terms of the higher mutational frequency and related function of seed genes in this subtype. These genes were called the “scored gene”. When a patient harbored one “scored gene” mutation, one score was awarded. Adding up the scores of each molecular subtype, remaining patients were classified based on the highest score in corresponding subtype.

Correlation between gene mutations and signaling pathways was calculated by R package *ggcorrplot* (version 0.1.3). *TNFAIP3*, *SOCS1* and *CIITA*, which represent three major molecular alterations in CD30+DLBCL, were defined as the “core seed gene”. There were totally 36 mutated genes involved in subtype establishment. Based on Gene Ontology database, these 36 mutated genes can be assigned to biological processes including immune response, methylation and acetylation, as well as oncogenic pathways TNFR-related NF-κB, JAK-STAT, and BCR-MAPK analyzed by R package *clusterProfiler* (version 3.16.1) (supplementary Table 6). As a result, 162 out of 172 (94.2%) CD30+DLBCL with DNA sequencing data were classified into three molecular subtypes.

5. RNA sequencing and bioinformatics analysis

Total RNA was extracted from frozen tumor tissue by Trizol and RNeasy Mini Kit (Qiagen). For RNA sequencing, RNA was purified using Ribo-Zero rRNA Removal Kits (Illumina). RNA concentration and integrity were verified using NanDrop and Agilent 2100 Bioanalyzer, respectively. Sequencing library was constructed using TruSeq RNA Samples Preparation Kit (Illumina). Qubit (Thermo Fisher Scientific) was used to quantify concentration of the resulting sequencing libraries, whereas the size distribution was analyzed using Agilent BioAnalyzer 2100 (Agilent). After library validation, clusters were generated by Illumina cBOT cluster generation system with HiSeq PE Cluster Kits (Illumina). Paired-end sequencing was performed using an Illumina HiSeq system following Illumina-provided protocols for 2×150 paired-end sequencing. Transcript counts table files were generated by the HTSeq using the GENCODE annotation database and processed with the BAM files generated by Hisat2.

RNA sequencing was performed in 385 patients, including 244 patients from our previous report^8^, and 141 patients newly added. Bioinformatic analyses were performed by r 3.6.1, using R package “sva” to remove batch effect. Limma (version 3.34.9) were used to normalize the raw reads and obtained differentially expressed genes (DEGs). Cell-of-origin (COO) group was determined as previously reported^9^. Gene Set Enrichment Analysis (GSEA) was performed using the GSEA (v2.2.3, http://software.broadinstitute.org/gsea/downloads.jsp) with MSigDB-curated gene sets (c2.cp.kegg.v6.2.symbols.gmt) and (c5.all.v7.1.symbols.gmt)^10^. Tumor microenvironment was analyzed using ImmuneCellAI (http://bioinfo.life.hust.edu.cn/web/ImmuCellAI/)^11^. Detailed sequencing protocols and bioinformatic principles were shown in the supplementary Methods.

6. Cell lines and reagents

GCB subtype B-lymphoma cell line SU-DHL-4 (obtained from American Type Culture Collection, Manassas, VA, USA) was grown in RPMI-1640 medium, and ABC subtype OCI-LY10 (kindly provided by Huang CX) was grown in IMDM, supplemented with 10% heat-inactivated fetal bovine serum and 1% penicillin/streptomycin (15140122, Gibco, Carlsbad, CA, USA) in a humidified atmosphere containing 95% air-5% CO_2_ at 37°C.

7. Cell transfection

Cell lines was transfected with viral particles (~5 × 10^8^ units/ml) containing purified plasmids expressing pGV367/GFP/Puro (vector), pGV367/GFP/Puro-*TNFAIP3* (NM_006290, wild-type, wt), pGV367/GFP/Puro-*TNFAIP3* (NM_006290, L147Q), pGV367/GFP/Puro-*SOCS1* (NM_003745, wild-type, wt), pGV367/GFP/Puro-*SOCS1* (NM_003745, Q175H), pGV367/GFP/Puro-*CIITA* (NM_000246, wild-type, wt), pGV367/GFP/Puro-*CIITA* (NM_000246, L807R), pGV248/GFP/Puro (Scramble), pGV248/GFP/Puro sh*TNFAIP3*, pGV248/GFP/Puro sh*SOCS1* and pGV248/GFP/Puro-sh*CIITA* using lipofectamine 2000 (11668019, Invitrogen, Carlsbad, CA, USA) according to the manufacturer’s protocol (MOI=50). The stably transduced clones were selected by green fluorescence protein using flow cytometry. The shRNA sequences were listed in supplementary Table 7.

8. Quantitative real-time PCR (qRT-PCR)

The total RNA was extracted using Trizol reagent and reverse transcribed using a PrimeScript RT Reagent Kit with gDNA Eraser for quantitative RT-PCR (RR047A, TaKaRa, Japan). Quantitative RT PCR was performed using SYBR Premix Ex TaqTM II (RR820A, TaKaRa) and ABI ViiA7 (Applied Biosystems, Bedford, MA, USA) following the manufacturer’s instructions. Relative quantification was calculated using the 2−ΔΔCT methods. The primers are listed in supplementary Table 8.

9. Western blot

Cells were lysed in 200 μl lysis buffer (0.5 M Tris-HCl, pH 6.8, 2 mM EDTA, 10% glycerol, 2% SDS, and 5% β-mercaptoethanol). Protein lysates (20 μg) were electrophoresed on 10% SDS polyacrylamide gels and transferred to nitrocellulose membranes. Membranes were blocked with 5% non-fat dried milk and incubated overnight at 4 °C with the appropriate primary antibodies, followed by a horseradish peroxidase-conjugated secondary antibody. The immunocomplexes were visualized using a chemiluminescence phototope-horseradish peroxidase Kit (Cell Signaling Technologies, Danvers, MA, USA). The antibodies are listed in supplementary Table 9.

10. Flow cytometry

Antibody used for testing CD30 was PE anti-CD30 (550041, BD Biosciences, USA). Flow cytometry data were collected by a FACS Calibur cytometer (BD Biosciences) and the median fluorescent intensity (MFI) was analyzed by FlowJo software (version 10.4).

11. Cell Viability

Cells (2×10^5^/ml) were seeded in 96-well plates and incubated with indicated concentration of reagents. Cell growth was assessed by CCK8 (1:10, Dojindo, Kumamoto, Japan) and the absorbance was measured at 450 nm by spectrophotometry. The percentage of cell growth inhibition was calculated as treated cells divided by untreated cells.

12. Statistical Analysis

Baseline characteristics of patients were analyzed using Fisher's exact tests or two-sided χ2 test. Differences among three subtypes were assessed by Mann-Whitney U-test. Correlation analysis was assessed by Spearman’s rank correlation. Progression-free survival (PFS) was measured from the date of diagnosis to the date when disease progression/relapse was recognized or the date of last follow-up. Overall survival (OS) was calculated from the date of diagnosis to the date of death or the date of last follow-up. Survival functions were estimated using the Kaplan-Meier method and compared by log-rank test. Statistical significance was defined as *P*<0.050. All statistical analysis was carried out using Statistical Package for the Social Sciences (SPSS) 25.0 software (SPSS Inc., Chicago, IL, USA) and R studio (version 3.6.1).

**Figure S1. CD30 expression in DLBCL.**

(A) Flowchart of the patient selection and methods. A total of 1122 patients with de novo DLBCL had immunohistochemical results of CD30, and 1048 patients were enrolled. DNA sequencing and RNA sequencing were available in 636 and 385 patients, respectively. CD30+DLBCL were divided into NM, JA, and IB subtypes. (B) Correlation between CD30 protein expression by immunohistochemistry and CD30 transcripts by RNA sequencing in DLBCL. (C) Semi-quantitative immunohistochemistry of CD30 expression in DLBCL.

**Figure S2. Survival analysis in DLBCL according to CD30 expression.**

Kaplan-Meier curves of progression-free survival (A) and overall survival (B) in DLBCL according to CD30 expression.

**Figure S3. Characteristics of molecular subtypes of CD30+DLBCL.**

(A) Genomic analysis distinguished three molecular subtypes of CD30+DLBCL. (B) Correlation of gene mutations in CD30+DLBCL (left panel) and correlation of signaling pathways in CD30+DLBCL (right panel). (C) Semi-quantitative IHC of CD30 expression in three molecular subtypes. (D) CD30 transcripts by RNA sequencing in three molecular subtypes. (E) EBER expression in three molecular subtypes.

**Figure S4. Gene expression signature and tumor microenvironment in CD30+DLBCL molecular subtypes.**

(A) Profile of signaling pathways according to CD30+DLBCL subtypes. Columns represent CD30+DLBCL patients. Heatmap of gene clustering is indicated at the bottom and related pathways are indicated at the top. (B) Correlation between Tfh score and expression of CCR6, CCL21, and CXCR4 (left panel). Correlation between CD4+T score and expression of CCR6, CCL21, and CXCR4 (middle panel). Correlation between DC score and expression of immune checkpoints PD-L1, TIM3 and LAG3 (right panel).

**Figure S5.** **B-lymphoma cell models of CD30+DLBCL.**

(A) Mutational locus of *TNFAIP3*/*SOCS1*/*CIITA* in CD30+DLBCL. Plots were created using cBioPortal’s Mutation Mapper Tool (https://www.cbioportal.org/mutation_mapper). (B) Expression of *CD30*/*TNFAIP3*/*SOCS1*/*CIITA* in OCI-LY10 and SU-DHL-4 cells. Error bars denote SD of three experiments. (C) Validation of transfection efficiency by quantitative real-time PCR of OCI-LY10 (left panel) and SU-DHL-4 (right panel) cells. (D) Validation of transfection efficiency by western blot of OCI-LY10 (left panel) and SU-DHL-4 (right panel) cells.


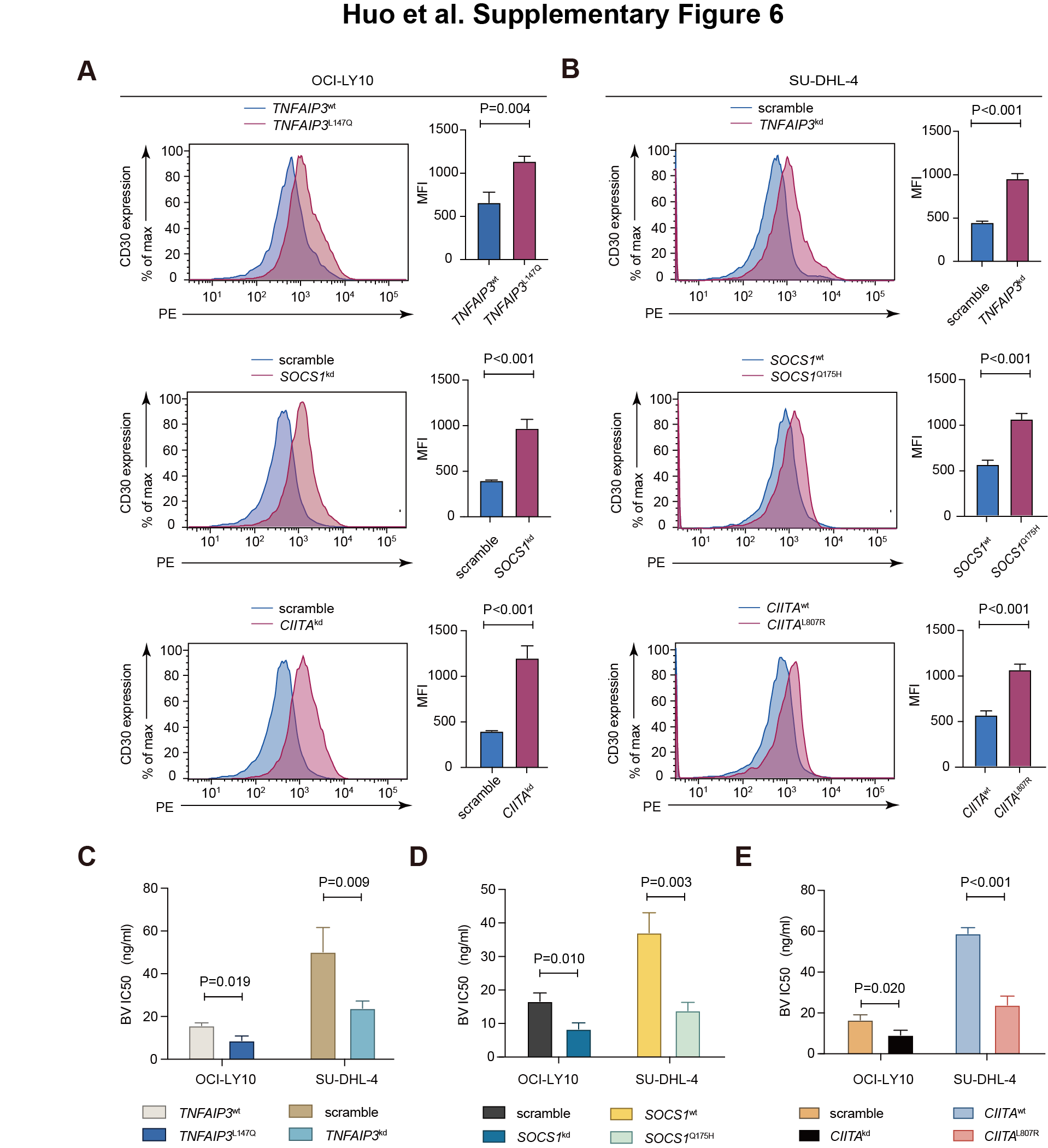


**Figure S6. B-lymphoma cell models and targeted therapy of CD30+DLBCL molecular subtypes.**

(A) CD30 expression was upregulated in *TNFAIP3*^kd^, as compared to scramble, with similar pattern in *SOCS1*^Q175H^ to *SOCS1*^wt^, and *CIITA*^L807R^ to *CIITA*^wt^ in OCI-LY10 cells. (B) CD30 expression was upregulated in *TNFAIP3*^L147Q^, *SOCS1*^kd^, *CIITA*^kd^, as compared to *TNFAIP3*^wt^ and scramble, respectively in SU-DHL-4 cells. (C) Half maximal inhibitory concentration (IC50) of OCI-LY10 cells transfected with *TNFAIP*3^wt^ or *TNFA*IP3^L147Q^ (left panel) and SU-DHL-4 cells transfected with *TNFAIP3* shRNA or scramble (right panel) treated with brentuximab vedotin. (D) IC50 of OCI-LY10 cells transfected with *SOCS1* shRNA or scramble (left panel) and SU-DHL-4 cells transfected with *SOCS1*^wt^ or *SOCS1*^Q175H^ (right panel) treated with brentuximab vedotin. (E) IC50 of OCI-LY10 cells transfected with *CIITA* shRNA or scramble (left panel) and SU-DHL-4 cells transfected with *CIITA*^wt^ or *CIITA*^L807R^ (right panel) treated with brentuximab vedotin. All the error bars denote SD of three experiments.

**Table S1. Clinical and pathological characteristics of DLBCL patients.**

|  | Overall (n=1048) n (%) | CD30+ (n=253) n (%) | CD30- (n=795) n (%) | P value |
| --- | --- | --- | --- | --- |
| Age>60 | 484 (46) | 112 (44) | 372 (47) | 0.515 |
| Gender, male | 580 (55) | 138 (55) | 442 (56) | 0.772 |
| ECOG, 2-4 | 126 (12) | 32 (13) | 94 (12) | 0.739 |
| Ann Arbor stage, III-IV | 536 (51) | 143 (57) | 393 (49) | 0.051 |
| ≥2 extranodal sites | 301 (29) | 73 (29) | 228 (29) | 1.000 |
| Elevated LDH | 541 (52) | 152 (60) | 389 (49) | 0.002 |
| IPI risk group |  |  |  | 0.040 |
| 0-1 | 478 (46) | 99 (39) | 379 (48) |  |
| 2 | 205 (20) | 52 (21) | 153 (19) |  |
| 3 | 200 (19) | 62 (25) | 138 (17) |  |
| 4-5 | 165 (16) | 40 (16) | 125 (16) |  |
| Non-GCB (n=958) | 606 (63) | 160 (69) | 446 (61) | 0.042 |
| DE (n=796) | 197 (25) | 45 (23) | 152 (25) | 0.506 |
| EBER+ (n=765) | 54 (7) | 26 (14) | 28 (5) | <0.001 |
| DH (n=634) | 28 (4) | 6 (4) | 22 (5) | 0.664 |

Abbreviations: ECOG, Eastern Cooperative Oncology Group; LDH, lactate dehydrogenase; GCB, germinal center B-cell; DE, double expression; EBER, Epstein-Barr virus-encoded RNA; DH, double hit

**Table S2. Clinical and pathological characteristics between CD30+DLBCL with CD30 level over 20% and CD30 level ranged from 1% to 20%**

| ­ | CD30+DLBCL (n=253)  n (%) | CD30≥20% (n=118)  n (%) | 1%≤CD30<20% (n=135)  n (%) | P value |
| --- | --- | --- | --- | --- |
| Age>60 | 112 (44) | 67 (56) | 66 (49) | 0.128 |
| Gender, male | 138 (55) | 138 (55) | 72 (53) | 0.705 |
| ECOG, 2-4 | 32 (13) | 15 (13) | 17 (13) | 1.000 |
| Ann Arbor stage, III-IV | 143 (57) | 65 (55) | 79 (59) | 0.612 |
| ≥2 extranodal sites | 73 (29) | 30 (25) | 44 (33) | 0.215 |
| Elevated LDH | 152 (60) | 70 (59) | 83 (62) | 0.701 |
| IPI risk group |  |  |  | 0.316 |
| 0-1 | 99 (39) | 48 (40) | 51 (38) |  |
| 2 | 52 (21) | 29 (24) | 23 (17) |  |
| 3 | 62 (25) | 27 (23) | 36 (27) |  |
| 4-5 | 40 (16) | 15 (13) | 25 (19) |  |
| Non-GCB (n=232) | 160 (69) | 75 (70) | 85 (69) | 0.888 |
| DE (n=198) | 45 (23) | 20 (23) | 25 (21) | 0.605 |
| EBER+ (n=192) | 26 (14) | 15 (18) | 11 (10) | 0.139 |
| DH (n=169) | 6 (4) | 4 (6) | 2 (2) | 0.403 |

Abbreviation: ECOG, Eastern Cooperative Oncology Group; LDH, lactate dehydrogenase; GCB, germinal center B-cell; DE, double expression; EBER, Epstein-Barr virus-encoded RNA; DH, double hit

**Table S3. Clinical and pathological characteristics of CD30+DLBCL molecular subtypes**

|  | NM subtype  (n = 61) n (%) | JA subtype  (n = 56) n (%) | IB subtype  (n = 45) n (%) | P value |
| --- | --- | --- | --- | --- |
| Age>60 | 33 (54) | 19 (34) | 22 (49) | 0.083 |
| ECOG, 2-4 | 5 (8) | 6 (11) | 9 (20) | 0.190 |
| Ann Arbor stage, III-IV | 36 (59) | 35 (63) | 21 (47) | 0.252 |
| ≥2 extranodal sites | 15 (25) | 22 (39) | 16 (36) | 0.215 |
| Elevated LDH | 35 (57) | 36 (64) | 31 (69) | 0.475 |
| IPI risk group |  |  |  | 0.079 |
| 0-1 | 21 (34) | 21 (38) | 17 (38) |  |
| 2 | 15 (25) | 7 (13) | 10 (22) |  |
| 3 | 19 (31) | 18 (32) | 6 (13) |  |
| 4-5 | 6 (10) | 10 (18) | 12 (27) |  |
| Non-GCB (n=156) | 39 (67) | 38 (69) | 31 (72) | 0.861 |
| DE (n=158) | 10 (17) | 18 (33) | 9 (21) | 0.116 |
| EBER+ (n=148) | 7 (13) | 7 (14) | 4 (9) | 0.775 |
| DH (n=137) | 1 (2) | 3 (7) | 0 (0) | 0.206 |

Abbreviation: ECOG, Eastern Cooperative Oncology Group; LDH, lactate dehydrogenase; GCB, germinal center B-cell; DE, double expression; EBER, Epstein-Barr virus-encoded RNA; DH, double hit

**Table S4. Univariate and multivariate analysis for progression-free survival and overall survival in CD30+DLBCL**

|  | Univariate analysis | | | |  | Multivariate analysis | | | |
| --- | --- | --- | --- | --- | --- | --- | --- | --- | --- |
| Characteristics | progression-free survival | | overall survival | | | progression-free survival | | overall survival | |
|  | P value | HR (95%CI) | P value | HR (95%CI) |  | P value | HR (95%CI) | P value | HR (95%CI) |
| Age>60 | 0.660 | 1.166(0.589-2.308) | 0.22 | 1.784(0.707-4.503) |  | 0.713 | 0.872(0.419-1.814) | 0.34 | 1.631(0.598-4.448) |
| ECOG, 2-4 | 0.041 | 2.379(1.035-5.470) | 0.115 | 2.450(0.804-7.467) |  | 0.11 | 2.069(0.848-5.046) | 0.264 | 2.040(0.585-7.120) |
| Ann Arbor stage, III/IV | 0.003 | 3.117(1.482-6.558) | 0.135 | 2.128(0.790-5.729) |  | 0.008 | 3.081(1.334-7.116) | 0.120 | 2.340(0.802-6.825) |
| ≥2 extranodal sites | 0.091 | 1.867(0.905-3.849) | 0.971 | 1.021(0.333-3.132) |  | 0.926 | 0.960(0.403-2.288) | 0.746 | 0.806(0.218-2.973) |
| Elevated LDH | 0.227 | 1.534(0.766-3.074) | 0.999 | 0.999(0.391-2.554) |  | 0.741 | 1.143(0.517-2.523) | 0.539 | 0.711(0.240-2.109) |
| JA subtype | 0.009 | 1.221(2.213-4.010) | 0.001 | 4.130(1.749-9.752) |  | 0.012 | 2.162(1.188-3.932) | <0.001 | 6.372(2.539-­15.994) |

Abbreviation: CI: confidence interval; ECOG, Eastern Cooperative Oncology Group; HR: hazard ratio; LDH, lactate dehydrogenase**­Table S5. Reagent and resource for immunohistochemistry**

| Biomarker | Antibody | Derived clone | Company | Cutoff value |
| --- | --- | --- | --- | --- |
| CD30 | F084901-2 | Ber-H2 | DAKO | 1% |
| CD10 | N.D. | 56C6 | DAKO | 30% |
| BCL6 | IS62530-2 | PG-B6p | DAKO | 30% |
| MUM1 | IS64430-2 | MUM1p | DAKO | 30% |
| BCL2 | N.D. | 124 | DAKO | 50% |
| MYC | ZA-0555 | EP121 | ZS biotechnologies, Ltd, Beijing, China | 40% |
| Ki-67 | F726801-8 | MIB-1 | DAKO | 85% |
| EBER | N.D. | 05278660001 | Roche | 20% |

Abbreviation: EBER, Epstein-Barr virus-encoded RNA

**Table S6. Genes in different pathways**

| **NF-κB signaling pathway** | | | | | | | | | |
| --- | --- | --- | --- | --- | --- | --- | --- | --- | --- |
| *TNFAIP3* | *TNFRSF14* | *CD70* | *NFKBIE* | *TBL1XR1* |  |  |  |  |  |
| **Methylation** | | | | | | | | | |
| *ARID1A* | *TET2* | *HIST1H1C* | *HIST1H1E* | *KMT2C* | *KMT2D* |  |  |  |  |
| **JAK-STAT signaling pathway** | | | | | | | | | |
| *SOCS1* | *CD58* | *STAT6* | *BCL6* | *EBF1* | *DUSP2* | *MYC* | *PIM1* | *ATM* | *NOTCH1* |
| **Acetylation** | | | | | | | | | |
| *CREBBP* | *EP300* |  |  |  |  |  |  |  |  |
| **Immune response** | | | | | | | | | |
| *CIITA* | *B2M* | *IRF4* | *IRF8* | *BTG1* | *BTG2* | *DDX3X* |  |  |  |
| **BCR-MAPK signaling pathway** | | | | | | | | | |
| *LYN* | *MYD88* | *CD79B* | *PTPN6* | *TSC2* | *SGK1* | *GNA13* |  |  |  |

Abbreviation: BCR: B-cell receptor

**Table S7. Sequences of shRNA**

|  | *TNFAIP3* | *SOCS1* | *CIITA* |
| --- | --- | --- | --- |
| shRNA1 | CGGCTATGACAGCCATCATTT | GGTAGCACACAACCAGGTGGC | AGGGCCTGAGCAAGGACATTT |
| shRNA2 | GCACCGATACACACTGGAAAT | GTGGCAGCCGACAATGCAGTC | GGCTACCTGGAGCTTCTTAAC |
| shRNA3 | CCAGGATGTTACCAGGACATT | GCCGACAATGCAGTCTCCACA | GGGTCTCCAGTATATTCATCT |

**Table S8. Sequences of primers for RT-PCR**

| Name | Forward | Reverse |
| --- | --- | --- |
| *TNFRSF8* | GCTGTCAGGAGGTGCTGTTAC | GTAGGCCTCTGTGGGCACT |
| *TNFAIP3* | TCCTCAGGCTTTGTATTTGAGC | TGTGTATCGGTGCATGGTTTTA |
| *SOCS1* | TTTTCGCCCTTAGCGTGAAGA | GAGGCAGTCGAAGCTCTCG |
| *CIITA* | CCTGGAGCTTCTTAACAGCGA | TGTGTCGGGTTCTGAGTAGAG |

**Table S9. Reagents and resources for western blot**

| Antibody | Identifier | Company |
| --- | --- | --- |
| Primary antibody | | |
| TNFAIP3 | #5630 | Cell Signaling Technologies |
| SOCS1 | #68631 | Cell Signaling Technologies |
| CIITA | 55099-1-AP | Proteintech |
| Secondary antibody | | |
| goat anti-mouse-IgG | #7076 | Cell Signaling Technologies |
| goat anti-rabbit-IgG | #7074 | Cell Signaling Technologies |

**References**

1. Swerdlow SH, Campo E, Pileri SA, Harris NL, Stein H, Siebert R, et al. The 2016 revision of the World Health Organization classification of lymphoid neoplasms. Blood. 2016;127(20):2375-90.

2. A predictive model for aggressive non-Hodgkin's lymphoma. N Engl J Med. 1993;329(14):987-94.

3. Jacobsen ED, Sharman JP, Oki Y, Advani RH, Winter JN, Bello CM, et al. Brentuximab vedotin demonstrates objective responses in a phase 2 study of relapsed/refractory DLBCL with variable CD30 expression. Blood. 2015;125(9):1394-402.

4. Ward J, Berrien-Elliott MM, Gomez F, Luo J, Becker-Hapak M, Cashen AF, et al. Phase I/Dose Expansion Trial of Brentuximab vedotin/Lenalidomide in Relapsed or Refractory Diffuse Large B-cell Lymphoma. Blood. 2021.

5. Hans CP, Weisenburger DD, Greiner TC, Gascoyne RD, Delabie J, Ott G, et al. Confirmation of the molecular classification of diffuse large B-cell lymphoma by immunohistochemistry using a tissue microarray. Blood. 2004;103(1):275-82.

6. Hu S, Xu-Monette ZY, Tzankov A, Green T, Wu L, Balasubramanyam A, et al. MYC/BCL2 protein coexpression contributes to the inferior survival of activated B-cell subtype of diffuse large B-cell lymphoma and demonstrates high-risk gene expression signatures: a report from The International DLBCL Rituximab-CHOP Consortium Program. Blood. 2013;121(20):4021-31; quiz 250.

7. Zhao CX, Wen JJ, Fu D, Xu PP, Cheng S, Wang L, et al. Clinical and molecular features of Epstein-Barr virus-positive diffuse large B-cell lymphoma: Results in a multi-center trial. Clin Transl Med. 2021;11(9):e539.

8. Shen R, Xu PP, Wang N, Yi HM, Dong L, Fu D, et al. Influence of oncogenic mutations and tumor microenvironment alterations on extranodal invasion in diffuse large B-cell lymphoma. Clin Transl Med. 2020;10(7):e221.

9. Wright G, Tan B, Rosenwald A, Hurt EH, Wiestner A, Staudt LM. A gene expression-based method to diagnose clinically distinct subgroups of diffuse large B cell lymphoma. Proc Natl Acad Sci U S A. 2003;100(17):9991-6.

10. Subramanian A, Tamayo P, Mootha VK, Mukherjee S, Ebert BL, Gillette MA, et al. Gene set enrichment analysis: a knowledge-based approach for interpreting genome-wide expression profiles. Proc Natl Acad Sci U S A. 2005;102(43):15545-50.

11. Miao YR, Xia M, Luo M, Luo T, Yang M, Guo AY. ImmuCellAI-mouse: a tool for comprehensive prediction of mouse immune cell abundance and immune microenvironment depiction. Bioinformatics. 2021.
